# Infection deteriorating hepatitis B virus related acute-on-chronic liver failure: a retrospective cohort study

**DOI:** 10.1186/s12876-020-01473-y

**Published:** 2020-09-29

**Authors:** Xing-Ran Zhai, Jing-Jing Tong, Hong-Min Wang, Xiang Xu, Xiu-Ying Mu, Jing Chen, Zi-Feng Liu, Yu Wang, Hai-Bin Su, Jin-Hua Hu

**Affiliations:** 1 Peking University 302 Clinical Medical School, Beijing, China; 2 Liver Failure Treatment and Research Center, The Fifth Medical Center of Chinese PLA General Hospital, Beijing, China; 3 Medical School of Chinese PLA, Beijing, China

**Keywords:** Acute-on-chronic liver failure, Hepatitis B virus, Infections, Prognosis

## Abstract

**Background:**

Infection is common in acute-on-chronic liver failure (ACLF), which may worsen the clinical condition and prognosis. However, the characteristics of infection and its influence on prognosis in hepatitis B virus related ACLF (HBV-ACLF) as defined by the European Association for the Study of the Liver (EASL) have not been clarified. We aimed to investigate the characteristics of infection and its influence on mortality in patients with HBV-ACLF defined by EASL in China.

**Methods:**

We performed a retrospective cohort study in patients with HBV-ACLF defined by EASL in a single center from January 2015 to December 2017. These patients were divided into two groups with and without infection. The incidence, sites of infection, isolated strains, and risk factors associated with mortality were evaluated.

**Results:**

A total of 289 patients were included, among them 185 (64.0%) were diagnosed with an infection. The most common type of infection was pneumonia (55.7%), followed by spontaneous bacterial peritonitis (47.6%) and others. The gram-negative bacteria were the most frequent (58.3%). Patients with one, two, and three or more infection sites had a gradually increasing incidence of sepsis (*P* < 0.01), septic shock (*P* < 0.001), and ACLF-3 (*P* < 0.05). Also, patients with infection isolated one, two, and three or more strains showed a growing incidence of sepsis (*P* < 0.01) and septic shock (*P* < 0.001). Patients with infection showed a significantly higher 28-day mortality than those without (*P* < 0.01), especially in patients with ACLF-3. Infection was identified as an independent risk factor for 28-day mortality in all HBV-ACLF patients. Pneumonia and sepsis were identified as independent predictors of 28-day mortality for patients with infection.

**Conclusions:**

Infection is associated with severe clinical course and high mortality in HBV-ACLF defined by EASL. The increased number of infection sites or isolated strains was associated with the occurrence of sepsis and septic shock. Pneumonia and sepsis were independent predictors for mortality in HBV-ACLF patients with infection.

## Background

The definition of acute-on-chronic liver failure (ACLF) is different between the eastern and western countries. European Association for the Study of the Liver (EASL) defined ACLF as a severe clinical syndrome characterized by acute insult, organ failures (OFs), and high short-term mortality in patients with liver cirrhosi s[1]. In western countries, alcoholic liver disease is the most common cause of ACLF. However, in the east of Asia, hepatitis B virus (HBV) infection accounts for the overwhelming majority of etiology [2]. Patients with HBV-related ACLF (HBV-ACLF) are susceptible to infection due to impaired immune function, intestinal barrier dysfunction, and genetic predisposition [3]. Previous studies reported that infection resulted in the progression of ACLF as defined by EASL and even led to higher clinical severity scores and short-term mortality, [4–6] possibly due to the production and release of inflammatory cytokines that led to necrosis and apoptosis of hepatocyte [7]. However, the etiology of these patients is complex and high alcohol consumption accounts for the majority. As demonstrated in a study, [8] the characteristics of HBV-ACLF were markedly distinct from those of alcohol-related ACLF. HBV-ACLF usually manifesting higher clinical severity score, mortality rate, higher incidence of liver failure, lower incidence of kidney failure, and fewer trigger events. Thus, the influence of infection on the prognosis of patients with HBV-ACLF as defined by EASL is still poorly understood.

The objective of this study is to demonstrate the detailed features of infection in patients with HBV-ACLF defined by EASL and explore its influence on the clinical condition and 28-day mortality.

## Methods

### Ethical approval

This study accords with the ethical guidelines of the 1975 Declaration of Helsinki and was approved by the Ethical Committee of the Fifth Medical Center of Chinese People’s Liberation Army (PLA) General Hospital (No. 2019016D). Informed consent was waived due to the study was retrospectively designed and the data were anonymous.

### Patient selection

This cohort study consecutively retrospectively included 345 patients with HBV-ACLF hospitalized from January 2015 to December 2017 in the Fifth Medical Center of Chinese PLA General Hospital. Chronic hepatitis B was diagnosed by hepatitis B surface antigen and/or hepatitis B virus deoxyribonucleic acid (HBV-DNA) positivity for ≥6 months [9]. ACLF was diagnosed based on the criteria proposed by the EASL/the American Association for the Study of Liver Diseases [10–12]. The exclusion criteria were as follows: (1) Less than 18 years old; (2) Hepatocellular carcinoma or other malignant tumors; (3) Serious extra-hepatic diseases; (4) Received liver transplantation within 28 days; (5) Lost to follow-up within 28 days; (6) Hospital stay less than 48 h; (7) Pregnancy.

### Definitions related to infection

The diagnostic criteria for bacterial infection were listed as follows. (1) Spontaneous bacterial peritonitis (SBP): neutrophils count ≥250/mm^3^ in ascitic fluid. (2) Pneumonia: clinical manifestations of infection associated with imaging examination showing that new pulmonary infiltration. (3) Urinary tract infection (UTI): high white blood cell (WBC) count (> 10/field) was found in urinary sediment accompanied by positive culture results of urine or innumerable WBC per field with negative culture results of urine. (4) Bacteremia: positive blood culture. (5) Skin and soft tissue infection (SSTI): symptoms of infection, such as redness, inflation, high temperature, and pain on the skin. (6) Spontaneous bacterial empyema (SBE): neutrophils number in pleural fluid ≥250/mm^3^. (7) Infectious diarrhea: diarrhea with stool microscopic examination showing WBC or routine stool culture showing evidence of pathogenic microorganisms. (8) Cholangitis: right upper abdominal pain, cholestasis, or radiologic evidence of biliary obstruction. (9) Unproven infection: the existence of fever and leukocytosis needs antibiotic treatment with no recognizable sources [13–15].

The criteria used to define fungal infection were as follows. (1) Invasive candidiasis: detection of Candida spp. in the cultures of blood or from other normally aseptic body fluids. (2) Invasive aspergillosis: discovery of Aspergillus by direct laboratory inspection or culture of respiratory specimens when the radiological evidence was consistent with lung infection [16].

Bacteria/fungal infection was grouped as community-acquired (CA), healthcare-associated (HCA), and nosocomial (NS) infections. NS infection referred to an infection that occurred 48 h after hospitalization. HCA infection was defined as an infection that occurred within 48 h after hospitalization and met any of the following: (1) Hospitalization or hemodialysis clinic, or intravenous chemotherapy during the past one month; (2) Hospital stay for ≥2 days, or surgery in the past half a year; (3) Residence in a medical center or a long-term care facility. CA infection referred to an infection that occurred within 48 h after hospitalization and the patients did not satisfy any criteria for HCA infection [17].

Sepsis and septic shock was defined according to Sepsis-3 [18]. Sepsis can be identified by an increase of the sequential organ failure assessment (SOFA) score ≥ 2 points which is caused by a maladjusted host response to infection. Septic shock, a subset of sepsis in which profound circulatory, cellular, and metabolic abnormalities are associated with a higher risk of mortality than with sepsis alone, can be identified by a vasopressor requirement to keep mean arterial pressure (MAP) of 65 mmHg or higher and serum lactate level higher than 2 mmol/L in absent of hypovolemia [18].

### Clinical severity scores and HBV-ACLF grade

The formula for the model for end-stage liver disease (MELD) score was as follows: MELD score = 3.78 × ln [Total bilirubin (mg/dL)] + 11.2 × ln (INR) + 9.57 × ln [Creatinine (mg/dL)] + 6.43 [19]. The formula for MELDNa was as follows: MELDNa = MELD − Na − [0.025 × MELD ×(140 − Na)] + 140 [20]. Child-Turcotte-Pugh (CTP) score was the summation of fractions of the five indexes, including hepatic encephalopathy (HE), ascites, Total bilirubin (TBil), Albumin (Alb), and Prothrombin time prolongation, with 1–3 points for each index, a minimum score of 5, and a maximum score of 15 [21]. The OFs were evaluated based on the chronic liver failure consortium organ failure score (CLIF-C OFs) system, and chronic liver failure consortium acute-on-chronic liver failure score (CLIF-C ACLFs) was computed as follow: CLIF-C ACLFs = 10 × [0.33 × CLIF-C OFs + 0.04 × Age+ 0.63 × ln(WBC count)-2] [22]. The SOFA score was assessed as described [23].

ACLF was graded as per the EASL-chronic liver failure consortium acute-on-chronic liver failure in cirrhosis study [10]. ACLF grade-1 (ACLF-1) refers to the existence of single kidney failure, or brain failure accompanied by kidney dysfunction or another single OF accompanied by kidney/brain dysfunction; ACLF grade-2 (ACLF-2) refers to the existence of two OFs; ACLF grade-3 (ACLF-3) refers to the existence of three or more OFs.

### Statistical analysis

The data were analyzed using SPSS software (version 24.0, IBM Corporation, Armonk, NY, USA) and plotted using the GraphPad Prism software (version 7.00, GraphPad Software, Inc., La Jolla, CA, USA) and Medcalc software (version 15.2.2, Medcalc Software baba, Ostend, Belgium). Measurement data with normal distribution were expressed as mean ± standard deviation (SD), and the t-test was used for comparison between two groups or analysis of variance for comparison among three or more groups. Measurement data that did not conform to the normal distribution were represented by the median and interquartile range (IQR), and the Kolmogorov-Smirnov Z rank-sum test was used for comparison between two groups or Kruskal-Wallis Test for comparison among three or more groups. Enumeration data were expressed as number (%) and intergroup comparison was performed by the chi-square test. Cox proportional hazards regression model was used for univariate and multivariate analysis of 28-day prognostic independent risk factors. Survival curves were estimated by the Kaplan-Meier method and the comparison was performed with the log-rank test. The difference was considered statistically significant if *P* < 0.05.

## Results

### Baseline characteristics of patients

**Fig. 1:**
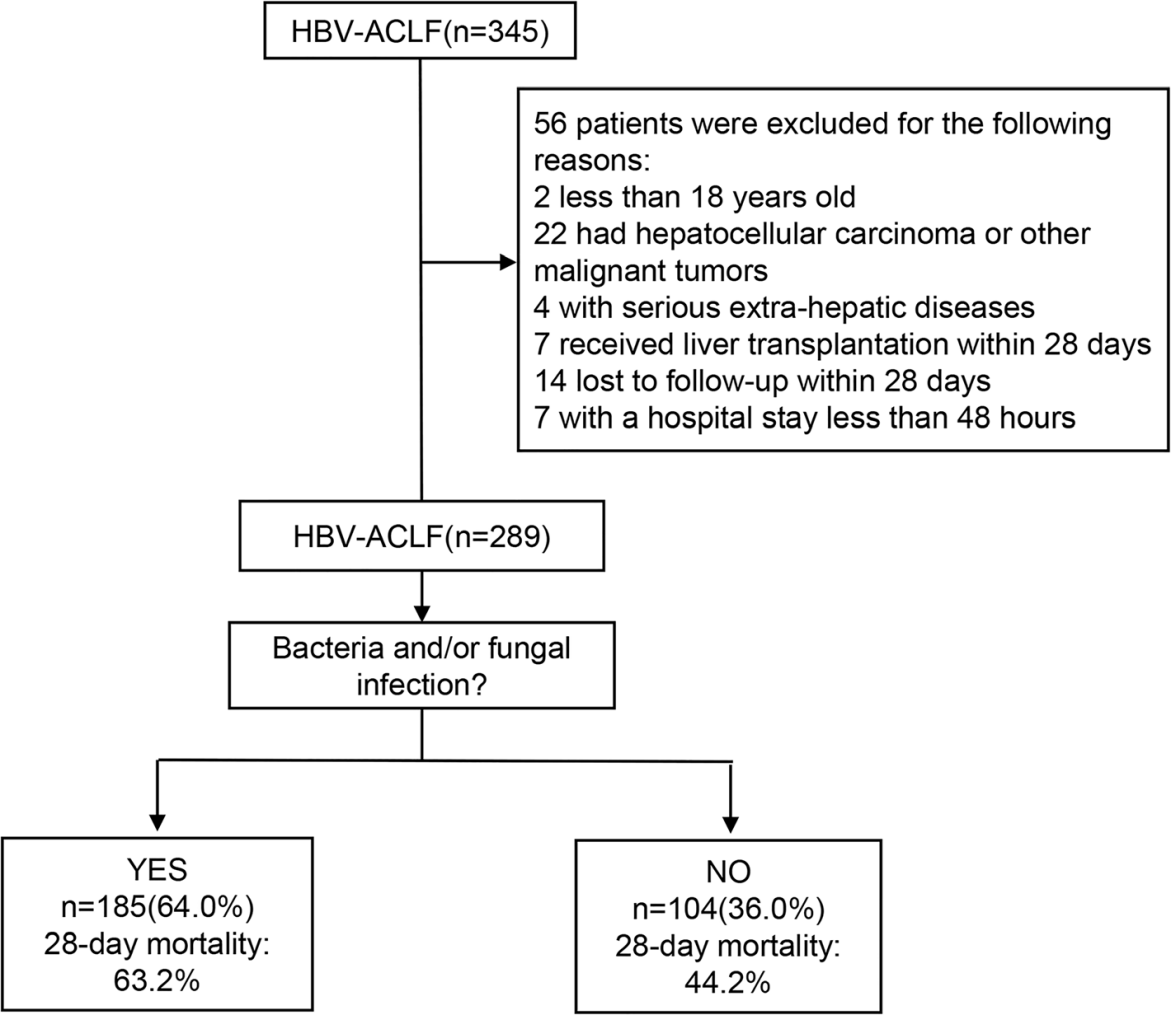
Flow chart of the patients with HBV-ACLF included and excluded from the study. HBV-ACLF: hepatitis B virus related acute-on-chronic liver failure

A total of 289 patients with HBV-ACLF were included in this study after excluding 56 patients according to the inclusion and exclusion criteria (Fig. 1), among them 236 (81.7%) were males and 53 (18.3%) were females. The mean age was 47.8 ± 10.9 years, range from 22 to 76 years old. A total of 185 (64.0%) patients developed bacterial or fungal infections during the study. Characteristics of HBV-ACLF patients with and without infection are shown in Table 1. Higher WBC count, serum creatinine (sCr), procalcitonin (PCT), lower MAP, Alb, higher incidence of hemorrhage, acute kidney injury (AKI), HE, kidney failure, brain failure, respiratory failure, circulation failure, systemic inflammatory reaction syndrome (SIRS), and higher clinical severity scores including CLIF-C ACLFs, CLIF-C OFs, SOFA scores were presented in patients with infection than non-infection patients.

**Table 1:** Baseline characteristics of HBV-ACLF patients with and without infection

Variables	Infection(*n* = 185)	No infection(*n* = 104)	*P*-value
Age (years)	48.0 ± 11.0	47.5 ± 10.6	0.731
Male (%)	151 (81.6)	85 (81.7)	0.982
BMI (kg/m^2^)	24.9 (22.4,27.7)	25.0 (22.1,28.1)	0.990
MAP (mmHg)	85 (79,95)	89 (81,96)	0.028
HBV-DNA level (IU/ml)			0.427
≤ 200	41 (22.3)	20 (19.2)	0.543
200–2 × 10^4^	42 (22.8)	33 (31.7)	0.098
2 × 10^4^–2 × 10^6^	57 (31.0)	28 (26.9)	0.469
≥ 2 × 10^6^	44 (23.9)	23 (22.1)	0.729
Laboratory data			
WBC count (10^9^/L)	9.7 (6.5,13.1)	7.4 (5.5,9.3)	< 0.001
Platelet count (10^9^/L)	64 (40,102)	71 (45,108)	0.201
Total bilirubin (μmol/L)	339 ± 162	366 ± 155	0.164
Albumin (g/L)	28.0 (24.0,30.5)	29.0 (26.3,32.0)	0.008
ALT (U/L)	102 (42,243)	96 (43,199)	0.881
AST (U/L)	135 (79,278)	128 (84,249)	0.855
ALP (U/L)	124 (100,169)	142 (110,171)	0.189
GGT (U/L)	50 (33,78)	49 (34,74)	0.736
INR	2.4 (1.9,2.8)	2.5 (2.0,2.7)	0.214
Creatinine (μmol/L)	135 (90,193)	111 (82,147)	0.015
Serum sodium (mmol/L)	133 (129,137)	133 (128,137)	0.997
HBeAg (%)	97 (53.0)	50 (48.1)	0.422
CRP (mg/L)	17.8 (9.1,53.9)	12.8 (8.4,20.8)	0.050
Procalcitonin (ng/ml)	1.2 (0.5,3.2)	0.6 (0.4,1.1)	< 0.001
Complications (%)			
Ascites	171 (92.4)	94 (90.4)	0.545
Hemorrhage	25 (13.5)	6 (5.8)	0.041
Acute kidney injury	107 (57.8)	43 (41.3)	0.007
HE	93 (50.3)	65 (62.5)	0.036
Organ failure (%)			
Liver failure	154 (83.2)	93 (89.4)	0.153
Coagulation failure	87 (47.0)	60 (57.7)	0.090
Kidney failure	54 (29.2)	17 (16.3)	0.015
Cerebral failure	33 (17.8)	6 (5.8)	0.004
Respiratory failure	24 (13.0)	3 (2.9)	0.005
Circulation failure	22 (11.9)	1 (1.0)	0.001
Prognostic score			
CLIF-C ACLFs	48 (44,53)	44 (40,48)	< 0.001
CLIF-C OFs	10 (10,12)	10 (9,11)	0.002
MELD	30 (26,34)	29 (25,32)	0.099
MELDNa	31 (28,36)	31 (27,34)	0.065
CTP	13 (12,13)	12 (11,13)	0.066
SOFA	9 (8,11)	8 (6,9)	< 0.001
ACLF grade (%)			
ACLF-1	62 (33.5)	36 (34.6)	0.849
ACLF-2	80 (43.2)	61 (58.7)	0.012
ACLF-3	43 (23.2)	7 (6.7)	< 0.001
SIRS (%)	57 (30.8)	12 (11.5)	< 0.001

Note: All data are expressed as number (%) or mean ± SD or median (IQR)

*Abbreviations*: *HBV-ACLF*: hepatitis B virus related acute-on-chronic liver failure; *BMI*: body mass index; *MAP*: mean arterial pressure; *HBV-DNA*: hepatitis B virus deoxyribonucleic acid; *WBC*: white blood cells; *ALT*: alanine aminotransferase; *AST*: aspartate aminotransferase; *ALP*: alkaline phosphatase; *GGT*: gamma-glutamyl transpeptidase; *INR*: international normalized ratio; *HBeAg*: hepatitis B virus envelope antigen; *CRP*: C-reactive protein; *HE*: hepatic encephalopathy; *CLIF-C ACLFs*: chronic liver failure consortium acute-on-chronic liver failure score; *CLIF-C OFs*: chronic liver failure consortium organ failure score; *MELD*: model for end stage liver disease; *CTP*: Child-Turcotte-Pugh; *SOFA*: sequential organ failure assessment; *SIRS*: system inflammatory reaction syndrome

### Characteristics of infection

The most common type of infection was pneumonia (55.7%, 103/185), followed by SBP (47.6%, 88/185), bacteremia (20.0%, 37/185), UTI (11.4%, 21/185), SBE (7.0%, 13/185), and other infections. The most common site of acquisition was NS infection in 94 (50.8%) patients, followed by HCA infection in 76 (41.1%), and CA infection in 15 (8.1%).

Positive culture results were detected in 120 among 185 patients with infection. Gram-positive bacteria, gram-negative bacteria, and fungi were identified in 42 (35.0%), 70 (58.3%), and 44 (36.7%) patients, respectively. Patients infected with fungi showed the highest 28-day mortality of 72.7% compared with 59.5 and 64.3% in those infected with gram-positive and gram-negative bacteria.

Two hundred and ten isolates were acquired from 120 patients with positive culture results. The most frequent isolated pathogen was gram-negative bacteria in 104 specimens (49.5%), among which the most common strain was *Escherichia coli* (18.6%), the most common source was ascites, followed by *Klebsiella pneumoniae* (11.4%), the most common source was ascites, and then Acinetobacter baumannii (5.7%), the most common source was sputum. Gram-positive bacteria were found in 53 (25.2%) isolates, among which the most frequent strain was *Enterococcus faecium* (9.0%), the most frequent source was ascites, followed by *Staphylococcus aureus* (3.3%), the most frequent source was sputum, and then *Staphylococcus epidermidis* (2.9%), the most frequent source was blood. Fungi were isolated in 53 (25.2%) specimens, among which Aspergillus fumigatus (11.0%) was the most prevalent, followed by *Candida albicans* (8.6%), the most prevalent source of both was sputum (Table 2).

**Table 2:** Distribution of bacteria and fungi and source of specimens

Microorganisms	Number	Constituent ratio(%)	Source of specimens(*n* = 210)					
			Ascites	Sputum	Urine	Blood	pleural fluid	Other secretion
Gram-positive bacteria(*n* = 53)								
*Enterococcus faecium*	19	9.0	9	2	3	2	1	2
*Staphylococcus aureus*	7	3.3	2	3	1	1	0	0
*Staphylococcus epidermidis*	6	2.9	2	0	0	4	0	0
Staphylococcus hominis	5	2.4	4	0	0	1	0	0
*Enterococcus faecalis*	4	1.9	2	0	2	0	0	0
Staphylococcus haemolyticus	2	1.0	0	1	0	1	0	0
Bacillus cereus	2	1.0	0	0	0	2	0	0
Streptococcus pneumoniae	1	0.5	0	0	0	1	0	0
Streptococcus oralis	1	0.5	1	0	0	0	0	0
Bacillus pumilus	1	0.5	0	0	0	1	0	0
Staphylococcus capitis	1	0.5	1	0	0	0	0	0
Methicillin-resistant coagulase negative staphylococcus	1	0.5	1	0	0	0	0	0
Lactobacillus fermenti	1	0.5	1	0	0	0	0	0
Bacillus vegetabile	1	0.5	0	0	0	1	0	0
Staphylococcus lentus	1	0.5	0	0	0	1	0	0
Gram-negative bacteria(*n* = 104)								
*Escherichia coli*	39	18.6	23	2	1	12	1	0
*Klebsiella pneumoniae*	24	11.4	8	6	2	6	1	1
Acinetobacter baumannii	12	5.7	2	7	0	2	1	0
Pseudomonas aeruginosa	8	3.8	1	6	0	1	0	0
Stenotrophomonas maltophilia	8	3.8	0	7	0	0	1	0
Klebsiella oxytoca	3	1.4	0	1	1	1	0	0
Chryseobacterium indologenes	2	1.0	2	0	0	0	0	0
Enterobacter aerogenes	1	0.5	0	1	0	0	0	0
Enterobacter asburiae	1	0.5	1	0	0	0	0	0
Raoultella ornithinolytica	1	0.5	0	0	0	1	0	0
Enterobacter cloacae	1	0.5	0	1	0	0	0	0
Raoultella planticola	1	0.5	1	0	0	0	0	0
Burkholderia cepacia	1	0.5	0	1	0	0	0	0
Pseudomonas putida	1	0.5	1	0	0	0	0	0
Flavobacterium meningosepticum	1	0.5	0	0	0	1	0	0
Fungi(*n* = 53)								
Aspergillus fumigatus	23	11.0	2	21	0	0	0	0
*Candida albicans*	18	8.6	2	12	4	0	0	0
*Candida tropicalis*	4	1.9	1	3	0	0	0	0
*Candida parapsilosis*	2	1.0	0	1	1	0	0	0
*Candida krusei*	2	1.0	0	1	1	0	0	0
*Candida glabrata*	1	0.5	0	1	1	0	0	0
Aspergillus flavus	1	0.5	0	1	0	0	0	0
Mucor	1	0.5	0	1	0	0	0	0

To study the characteristics of the infection-related index of different type of infection and different pathogens, WBC count, percentage of neutrophils, C-reactive protein (CRP), PCT, the incidence of SIRS, sepsis, septic shock, and ACLF grades were analyzed in patients with single-site infection and single-class pathogen infection. However, there was no statistically significant difference among them (Table 3).

**Table 3:** Characteristics of the infection-related index of different infection sites and pathogens

Variables	N	WBC(10^9^/L)	*N*(%)	CRP (mg/L)	PCT (ng/ml)	SIRS(%)	Sepsis(%)	Septic shock(%)	ACLF-1(%): ACLF-2(%): ACLF-3(%)	28-day mortality(%)
Type of infections										
Pneumonia alone	52	10.3 (7.3,13.6)	82 (72,89)	16.4 (11.0,54.7)	1.4 (0.8,4.4)	16 (30.8)	33 (63.5)	19 (36.5)	10 (19.2): 25 (48.1): 17 (32.7)	42 (80.8)
SBP alone	37	9.1 (5.8,13.1)	79 (67,85)	17.5 (8.8,51.2)	1.0 (0.5,1.6)	14 (37.8)	17 (45.9)	6 (16.2)	12 (32.4): 20 (54.1): 5 (13.5)	12 (32.4)
Bacteraemia alone	10	11.1 (6.6,15.5)	71 (65,82)	41.0 (24.5,59.2)	2.2 (1.0,4.6)	3 (30.0)	6 (60.0)	2 (20.0)	4 (40.0): 4 (40.0): 2 (20.0)	6 (60.0)
Others^a^	16	8.7 (4.8,14.9)	73 (62,82)	26.4 (9.4,68.6)	1.1 (0.5,5.0)	5 (31.3)	10 (62.5)	4 (25.0)	5 (31.3): 9 (56.3): 2 (12.5)	8 (50.0)
Number of infection sites										
One	115	9.9 (6.5,13.5)	79 (68,86)	18.9 (11.2,53.0)	1.4 (0.5,2.8)	38 (33.0)	66 (57.4)^**^	31 (27.0)^***^	31 (27.0): 58 (50.4): 26 (22.6)^*^	68 (59.1)
Two	50	9.2 (5.1,13.7)	81 (74,87)	19.0 (10.9,69.5)	1.5 (0.6,4.1)	17 (34.0)	39 (78.0)	24 (48.0)	22 (44.0): 18 (36.0):10 (20.0)	32 (64.0)
Three or more	20	12.9 (6.9,21.8)	81 (76,84)	55.6 (10.1,99.5)	0.9 (0.7,4.9)	8 (40.0)	17 (85.0)	14 (70.0)	9 (45.0): 4 (20.0): 7 (35.0)	17 (85.0)
Type of pathogens										
G- alone	42	8.9 (7.0,12.8)	79 (70,88)	29.3 (13.6,73.9)	1.6 (0.8,4.9)	14 (33.3)	25 (59.5)	13 (31.0)	18 (42.9): 18 (42.9): 6 (14.3)	25 (59.5)
G+ alone	26	10.7 (7.5,15.5)	79 (74,86)	34.0 (14.4,87.3)	1.5 (0.6,2.8)	10 (38.5)	16 (61.5)	5 (19.2)	8 (30.8): 12 (46.2): 6 (23.1)	15 (57.7)
Fungus alone	23	10.3 (5.7,15.1)	79 (69,88)	20.4 (9.3,55.0)	1.5 (0.9,4.5)	7 (30.4)	11 (47.8)	4 (17.4)	7 (30.4): 10 (43.5): 6 (26.1)	15 (65.2)
Number of strains										
One	86	9.7 (6.6,13.0)	79 (70,87)	27.6 (13.5,65.7)	1.5 (0.9,4.5)	29 (33.7)	49 (57.0)^**^	21 (24.4)^***^	30 (34.9): 39 (45.3): 17 (19.8)	52 (60.5)
Two	16	10.0 (6.0,14.8)	80 (72,88)	10.9 (7.2,71.3)	1.1 (0.5,2.8)	6 (37.5)	12 (75.0)	9 (56.3)	5 (31.3): 8 (50.0): 3 (18.8)	10 (62.5)
Three or more	18	12.6 (6.5,21.0)	82 (74,88)	55.2 (9.6,94.5)	1.2 (0.5,5.9)	6 (33.3)	18 (100.0)	17 (94.4)	5 (27.8): 5 (27.8): 8 (44.4)	14 (77.8)

Note: All data are expressed as number (%) or median (IQR)

*Abbreviations*: *N* number, *WBC* white blood cells, *N(%)* percentage of neutrophils, *CRP* C-reactive protein, *PCT* procalcitonin, *SIRS* system inflammatory reaction syndrome, *ACLF* acute-on-chronic liver failure, *SBP* spontaneous bacterial peritonitis, *G-* Gram-negative bacteria, *G+* Gram-positive bacteria

^a^Other infections included urinary tract infection alone (8): skin and soft tissue infection alone (1): infections diarrhea alone (2): spontaneous bacterial empyema alone (1): the undefined infection alone (4)

^*^P-value< 0.05

^**^P-value< 0.01

^***^P-value< 0.001

### The influence of the increasing number of infection sites and strains on sepsis and septic shock

One hundred and fifteen (62.2%) presented one infection site, 50 (27.0%) had two, and 20 (10.8%) had three or more in 185 HBV-ACLF patients with infection. Patients with one, two, three or more infection sites had a gradually increasing incidence of sepsis (57.4% vs 78.0% vs 85.0%, *P* < 0.01), septic shock (27.0% vs 48.0% vs 70.0%, *P* < 0.001), ACLF-3 (22.6% vs 20.0% vs 35.0%, *P* < 0.05) and 28-day mortality (59.1% vs 64.0% vs and 85.0%, *P* > 0.05)(Table 3).

One, two, three or more strains were isolated in 86 (71.7%), 16 (13.3%), and 18 (15.0%) patients, respectively, who showed a gradually increasing incidence of sepsis (57.0% vs 75.0% vs 100.0%, *P* < 0.01), septic shock (24.4% vs 56.3% vs 94.4%, *P* < 0.001), and 28-day mortality (60.5% vs 62.5% vs 77.8%, *P* > 0.05) (Table 3).

### The influence of infection on 28-day mortality in HBV-ACLF patients

**Fig. 2:**
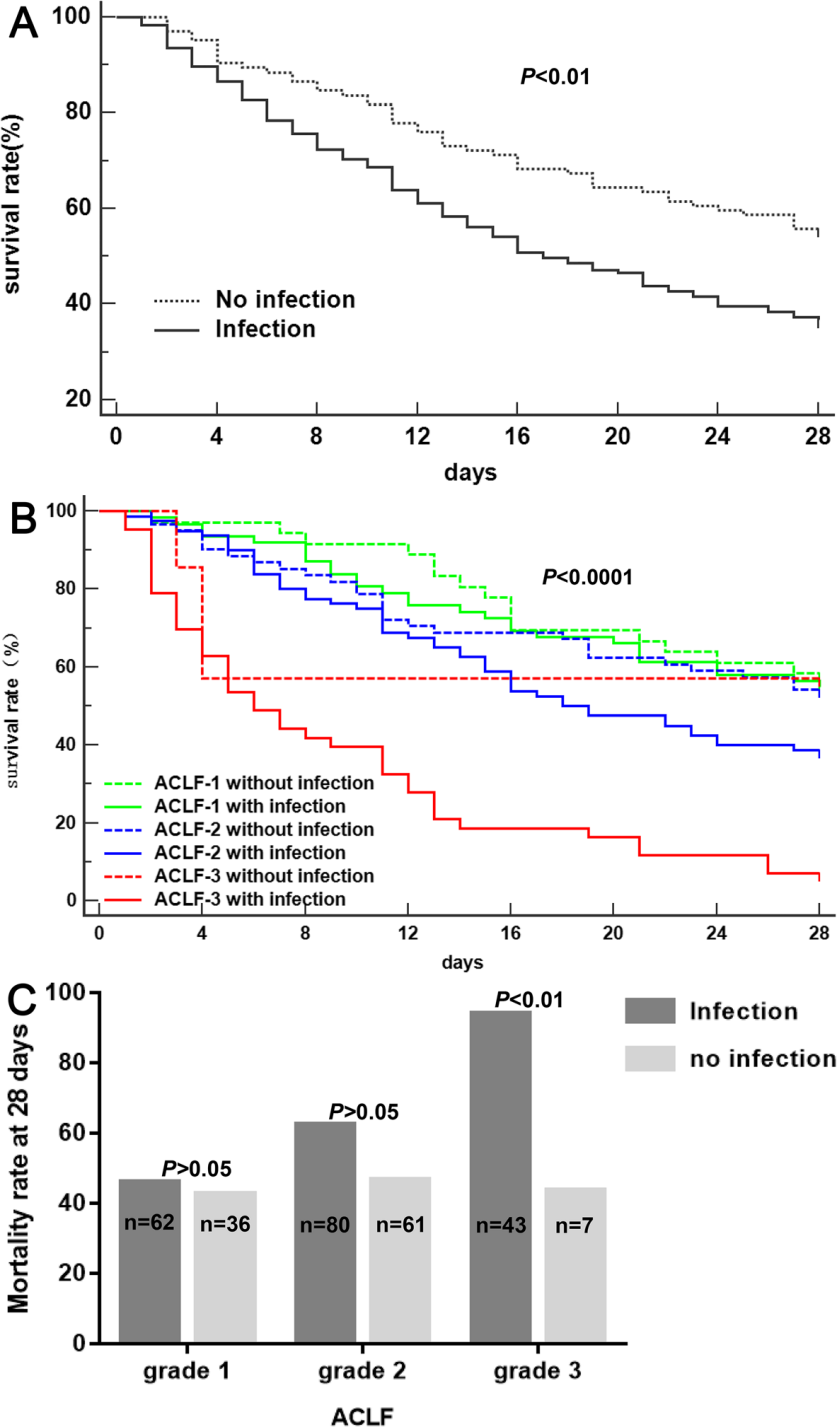
**a** Kaplan-Meier survival curves of patients with and without infection. **b** Kaplan-Meier survival curves of patients with different ACLF grades with and without infection. **c** The 28-day mortality rate of patients with different ACLF grades with and without infection

Patients with infection showed higher 28-day mortality than those without (63.2% vs 44.2%, *P* < 0.01) (Fig. 2 a). On univariate Cox analysis, the presence of infection, AKI, hemorrhage, HE, high WBC count, TBil, international normalized ratio (INR), creatinine, low serum sodium were associated with 28-day mortality. As creatinine and AKI had a strong correlation, creatinine was excluded in the multivariate Cox analysis to avoid collinearity. Multivariate Cox regression analysis showed that the presence of infection (HR = 1.515, 95% CI 1.071–2.144, *P* < 0.05) was an independent risk factor for 28-day mortality. Other independent risk factors for 28-day mortality included the presence of AKI (HR = 1.875, 95% CI 1.365–2.577, *P* < 0.001), HE (HR = 1.854, 95% CI 1.352–2.544, *P* < 0.001), high TBil (HR = 1.001, 95% CI 1.000–1.002, *P* < 0.05), and INR (HR = 1.730, 95% CI 1.472–2.035, *P* < 0.001) (Table 4).

**Table 4:** Predictors for 28-day mortality by Cox analysis in patients with HBV-ACLF

Predictors	Univariate analysis		Multivariate analysis	
	HR(95% CI)	*P*-value	HR (CI 95%)	*P*-value
Age (years)	1.001 (0.986 to 1.015)	0.934		
BMI (kg/m^2^)	1.022 (0.989 to 1.056)	0.201		
MAP (mmHg)	1.007 (0.992 to 1.022)	0.376		
Laboratory data				
WBC count(10^9^/L)	1.045 (1.020 to 1.071)	< 0.001		
Platelet count(10^9^/L)	0.999 (0.996 to 1.002)	0.416		
Total bilirubin (μmol/L)	1.001 (1.000 to 1.002)	0.072	1.001 (1.000 to 1.002)	0.019
Albumin(g/L)	0.992 (0.964 to 1.022)	0.599		
INR	1.694 (1.420 to 2.021)	< 0.001	1.730 (1.472 to 2.035)	< 0.001
Creatinine (μmol/L)	1.002 (1.000 to 1.003)	0.022		
Serum sodium (mmol/L)	0.977 (0.953 to 1.001)	0.062		
HBV-DNA level (IU/ml)				
≤ 200	1	0.109		
200–2 × 10^4^	0.779 (0.488 to 1.245)	0.297		
2 × 10^4^–2 × 10^6^	1.090 (0.705 to 1.685)	0.699		
≥ 2 × 10^6^	1.351 (0.867 to 2.107)	0.184		
Complications(%)				
Infection	1.725 (1.225 to 2.427)	0.002	1.515 (1.071 to 2.144)	0.019
Ascites	0.868 (0.502 to 1.501)	0.612		
Acute kidney injury	1.549 (1.134 to 2.116)	0.006	1.875 (1.365 to 2.577)	< 0.001
Haemorrhage	1.860 (1.205 to 2.871)	0.005		
HE	1.472 (1.295 to 1.674)	< 0.001	1.854 (1.352 to 2.544)	< 0.001

*Abbreviations*: *HBV-ACLF* hepatitis B virus related acute-on-chronic liver failure, *HR* hazard ratio, *BMI* body mass index, *MAP* mean arterial pressure, *WBC* white blood cells, *INR* international normalized ratio, *HBV-DNA* hepatitis B virus deoxyribonucleic acid, *HE* hepatic encephalopathy

Patients with ACLF-1, ACLF-2, and ACLF-3 complicate with infection showed a lower 28-day survival rate than those without infection (*P* < 0.0001) (Fig. 2 b). Among HBV-ACLF grade-3 patients, those with infection showed a significantly higher 28-day mortality than those without (93.0% vs 42.9%, *P* < 0.01). Patients with grade-1 and -2 showed the same tendency as grade-3 although there was no significant difference between infection and non-infection group (grade-1: 45.2% vs 41.7%, *P* = 0.737; grade-2: 61.3% vs 45.9%, *P* = 0.070) (Fig. 2 c).

### The influence of pneumonia and sepsis on 28-day mortality in HBV-ACLF patients with infection

Table 5 shows the predictors associated with 28-day mortality in the Cox regression analysis in 185 HBV-ACLF patients with infection. According to univariate analysis, the presence of pneumonia, SBP, ascites, AKI, HE, SIRS, sepsis, septic shock, elevated WBC, INR were significantly associated with 28-day mortality. As sepsis and septic shock had a strong correlation, septic shock was excluded in the multivariate Cox analysis. In multivariable Cox regression analysis, the presence of pneumonia (HR = 1.904, 95% CI 1.268–2.860, *P* < 0.01) and sepsis (HR = 2.166, 95% CI 1.372–3.421, *P* < 0.01) were identified as independent predictors for 28-day mortality. Other independent predictors included the presence of AKI (HR = 1.529, 95% CI 1.046–2.235, *P* < 0.05), HE (HR = 1.263, 95% CI 1.087–1.466, *P* < 0.01), and elevated INR (HR = 1.482, 95% CI 1.229–1.788, *P* < 0.001).

**Table 5:** Predictors for 28-day mortality by Cox analysis in HBV-ACLF patients with infection

Predictors	Univariate analysis		Multivariate analysis	
	HR(95% CI)	*P*-value	HR(95% CI)	*P*-value
Age (years)	0.996 (0.980 to 1.013)	0.676		
BMI (kg/m^2^)	1.028 (0.979 to 1.078)	0.268		
MAP (mmHg)	1.001 (0.985 to 1.018)	0.901		
Laboratory data				
WBC count (10^9^/L)	1.038 (1.010 to 1.066)	0.007		
Platelet count(10^9^/L)	0.998 (0.995 to 1.002)	0.306		
Total bilirubin (μmol/L)	1.000 (0.999 to 1.001)	0.498		
Albumin(g/L)	0.998 (0.967 to 1.030)	0.913		
INR	1.755 (1.455 to 2.118)	< 0.001	1.482 (1.229 to 1.788)	< 0.001
Creatinine (μmol/L)	1.001 (1.000 to 1.003)	0.105		
Serum sodium (mmol/L)	0.983 (0.955 to 1.011)	0.233		
HBV-DNA level (IU/ml)				
≤ 200	1	0.126		
200–2 × 10^4^	0.599 (0.341 to 1.051)	0.074		
2 × 10^4^–2 × 10^6^	0.864 (0.528 to 1.415)	0.562		
≥ 2 × 10^6^	1.151 (0.694 to 1.911)	0.586		
SIRS	1.658 (1.136 to 2.421)	0.009		
Type of infection(%)				
Pneumonia	2.315 (1.563 to 3.429)	< 0.001	1.904 (1.268 to 2.860)	0.002
SBP	0.623 (0.429 to 0.904)	0.013		
Bacteremia	1.260 (0.815 to 1.949)	0.299		
Number of infection sites				
One	1	0.132		
Two	1.184 (0.778 to 1.803)	0.430		
Three or more	1.718 (1.008 to 2.926)	0.046		
Number of isolated microorganisms				
Zero	1	0.638		
One	0.941 (0.625 to 1.418)	0.773		
Two	0.911 (0.456 to 1.819)	0.791		
Three or more	1.375 (0.749 to 2.524)	0.304		
Complications(%)				
Ascites	0.777 (0.585 to 1.031)	0.081		
Hemorrhage	1.447 (0.885 to 2.367)	0.141		
Acute kidney injury	1.456 (1.001 to 2.117)	0.049	1.529 (1.046 to 2.235)	0.028
HE	1.409 (1.214 to 1.636)	< 0.001	1.263 (1.087 to 1.466)	0.002
Sepsis(%)	2.740 (1.756 to 4.273)	< 0.001	2.166 (1.372 to 3.421)	0.001
Septic shock(%)	2.322 (1.612 to 3.345)	< 0.001		

*Abbreviations*: *HBV-ACLF* hepatitis B virus related acute-on-chronic liver failure, *HR* hazard ratio, *BMI* body mass index, *MAP* mean arterial pressure, *WBC* white blood cells, *INR* international normalized ratio, *HBV-DNA* hepatitis B virus deoxyribonucleic acid, *SIRS* system inflammatory reaction syndrome, *SBP* spontaneous bacterial peritonitis, *HE* hepatic encephalopathy

## Discussion

ACLF is a clinical syndrome with high short-term mortality. Usually, infection is not only the trigger of ACLF but also a common complication in patients with ACLF. Once an infection occurred, the mortality rate increased significantly [4, 5]. Excessive inflammation response induced by infection can cause multiorgan failure and death. Compared with the general population, infection is more prone to cause sepsis and septic shock. Therefore, it is vital to identify the characteristics of infection in the management of patients with ACLF. There are many studies about infection in patients with ACLF. But these studies were mainly carried out in western countries. It’s well known that the etiology is different between western and eastern countries. In eastern countries, HBV infection is the main cause of ACLF.

Our research is unique because the characteristics and influence of infection on the clinical condition and short-term prognosis were assessed in patients with HBV-ACLF defined by EASL in China. There are four main findings in our research. The first is that pneumonia is the most common form of infection in patients with HBV-ACLF. Gram-negative bacteria were the most frequent cultured microorganisms and *Escherichia coli* was predominant. Secondly, the infection had a negative influence on the short-term prognosis for HBV-ACLF patients. Thirdly, the increased number of infection sites or isolated strains was associated with an increased risk for sepsis and septic shock. Last but not the least, pneumonia and sepsis can serve as independent predictors for short-term mortality in HBV-ACLF patients with infection.

In our research, the most common type of infection was pneumonia, followed by SBP, bacteremia, UTI, SBE, and others. A previous study [5] in India reported that the most common site of infection was lungs in 172 (45.0%), followed by SBP in 81 (21.1%) and UTI in 58 (15.2%) in patients with ACLF. Another study [15] in China reported that the top three infection sites were the abdominal cavity, respiratory tract, and urinary tract in patients with HBV-ACLF. The results of the above studies were similar to that of our research.

One hundred and twenty patients had positive culture results in 185 patients with infection and 210 strains were isolated. Gram-negative bacteria accounted for the majority of all detected samples and were mostly *Escherichia coli* isolated from ascites. There have been studies [15, 24] reporting that gram-negative bacteria were the most frequent pathogen in patients with end-stage liver disease, which were basically in agreement with the present study, possibly owing to bacterial translocation caused by gut dysbiosis and increased permeability [3, 25]. Thus, early detection and prompt empirical antibiotic treatment are necessary for the management of HBV-ACLF patients with infection.

In our study, HBV-ACLF patients presented overall 28-day mortality of 56.4%, with a 28-day mortality of 63.2 and 44.2% in patients with and without infection, respectively. Infection was identified as an independent risk factor for 28-day survival in patients with HBV-ACLF. This negative impact was especially evident in patients with HBV-ACLF grade-3, in whom the 28-day mortality of patients with infection was significantly higher than those without. This result was different from that of a study conducted by Fernández et al, [4] which reported that ACLF grade-1 and grade-2 patients with infection showed lower 90-day survival than those without, but ACLF grade-3 with and without infections did not show differences in 90-day prognosis. In this study, more than half of the ACLF patients were caused by heavy drinking. The possible explanation for dissimilar results may be the difference in etiologies.

Sepsis refers to the development of organ dysfunction caused by infection. A recent study [26] showed that sepsis was identified as an independent predictor of in-hospital mortality and 28-day mortality using Cox regression analysis in patients with liver cirrhosis and bacterial/fungal infections. Septic shock represents severe circulatory disorders. In our study, increased infection sites and isolated strains were accompanied by an increased incidence of sepsis and septic shock, which undoubtedly complicated the clinical condition and make treatment more challenging. This phenomenon was rarely found in previous studies. Therefore, more attention should be paid to multiple types of infections to avoid deterioration of the clinical course.

Previous research [27] reported that pneumonia and sepsis were independent predictors for 30-day mortality in cirrhosis patients with ascites. Our study found that pneumonia and sepsis were identified as independent predictors for 28-day mortality in HBV-ACLF patients with infection. According to the related literature, tracheal intubations [28] and blood products transfusions [29] are considered to be risk factors for pneumonia. Thus, it is imperative to carefully monitor the signs of pneumonia and avoid the related risk factors for early prevention and timely treatment.

Additionally, it has been reported that ACLF patients with fungal infection had a high risk of death [30, 31]. A study performed by Verma et al [30] showed that ACLF patients with invasive fungal infections (IFI) had higher in-hospital mortality than those without IFI (76.9% vs 20%, *P* < 0.001). Lin et al [31] reported that the 12-week mortality was higher in HBV-ACLF patients with IFI as compared to those without (40.0% vs 12.1%, *P* < 0.001). Our study also found that patients with fungal infections had a higher mortality rate than those without (72.7% vs 53.5%, *P* < 0.05, see supplementary table). Besides pneumonia and sepsis, we also found that AKI, HE, and INR were independent predictors for 28-day mortality in HBV-ACLF patients with infection. A study performed by Zang et al [32] showed that the development of AKI was a predictor of 180-day survival in ACLF patients using the proportional sub-distribution hazards model. Besides, a recent study [33] showed that ACLF patients with AKI had a significantly higher 30-day and 90-day mortality than those without (79.6% vs 41.1, 82.7% vs 56.7%, *P* < 0.05). The results of the above researches are consistent with the results of our study. HE, one of the most common severe complications of ACLF, may cause brain edema and intracranial hypertension, which contribute to the progression of ACLF. INR reflects liver coagulation function and severity of liver necrosis.

Our study indeed had three main limitations. Firstly, the history of invasive manipulation and the use of glucocorticoids before infection were not clear in our database, which may be associated with the occurrence of infection. Secondly, this study was retrospectively designed, and it only involved a single center with relatively limited sample numbers, thus a prospective, multi-center study with a larger sample size on HBV-ACLF patients is necessary to further confirm the results of our study. Thirdly, the relation of HBV treatment status and HBV load to infection was not assessed.

## Conclusions

Pneumonia is the most common type of infection in HBV-ACLF patients. Gram-negative bacteria accounted for the most majority of cultured microorganisms, especially *Escherichia coli*. Infection was associated with increased mortality in HBV-ACLF patients. The increased incidence of sepsis and septic shock was significantly associated with an increased number of infection sites and cultured strains. Pneumonia and sepsis were independent predictors for short-term mortality in HBV-ACLF patients with infection. Clinical physicians should pay more attention to infections, especially pneumonia, sepsis, and multiple infections to avoid the progression of HBV-ACLF.

## Supplementary information

**Additional file 1 Supplementary table** Mortality at 28 days of HBV-ACLF patients with and without fungal infections.

## Data Availability

The datasets used and/or analyzed during the current study are available from the corresponding author on reasonable request.

## Abbreviations

ACLF: Acute-on-chronic Liver failure
HBV: Hepatitis B virus
HBV-ACLF: HBV-related ACLF
EASL: European Association for the Study of the Liver
PLA: People’s Liberation Army
HBV-DNA: Hepatitis B virus deoxyribonucleic acid
SBP: Spontaneous bacterial peritonitis
UTI: Urinary tract infection
WBC: White blood cell
SSTI: Skin and soft tissue infection
SBE: Spontaneous bacterial empyema
CA: Community-acquired
HCA: Healthcare-associated
NS: Nosocomial
SOFA: Sequential organ failure assessment
MAP: Mean arterial pressure
MELD: Model for end-stage liver disease
CTP: Child-Turcotte-Pugh
HE: Hepatic encephalopathy
TBil: Total bilirubin
Alb: Albumin
OFs: Organ failures
CLIF-C OFs: Chronic liver failure consortium organ failure score
CLIF-C ACLFs: Chronic liver failure consortium acute-on-chronic liver failure score
SD: Standard deviation
IQR: Interquartile range
sCr: Serum creatinine
PCT: Procalcitonin
AKI: Acute kidney injury
SIRS: Systemic inflammatory reaction syndrome
CRP: C-reactive protein
INR: International normalized ratio
IFI: Invasive fungal infections
