# Supplementary material for: Infection deteriorating hepatitis B virus related acute-on-chronic liver failure: a retrospective cohort study
Source: BMC Gastroenterol. 2020 Sep 29;20:320. doi: 10.1186/s12876-020-01473-y (PMC7526233; doi:10.1186/s12876-020-01473-y)
Supplement: Supplementary file 1 — Additional file 1 Supplementary table Mortality at 28 days of HBV-ACLF patients with and without fungal infections. [file 12876_2020_1473_MOESM1_ESM.docx]

| **Supplementary table** Mortality at 28 days of HBV-ACLF patients with and without fungal infections | | | |
| --- | --- | --- | --- |
| Patients | Number | 28-day mortality | P-value |
| Fungal infections | 44 | 32(72.7%) | 0.013 |
| Non-fungal infections | 245 | 131(53.5%) |  |

Note: All data are expressed as number or number (%).

Abbreviations: HBV-ACLF: hepatitis B virus related acute-on-chronic liver failure.
